# Herpes Simplex Virus Pseudotumor Masking as Gastric Malignancy

**DOI:** 10.14309/crj.0000000000000985

**Published:** 2023-03-08

**Authors:** Simran Kripalani, Jennifer Williams, Upasana Joneja, Preeti Bansal, Francis Spitz

**Affiliations:** 1 Temple University Hospital, Philadelphia, PA; 2 Cooper University Hospital, Camden, NJ

**Keywords:** HSV pseudotumor, HIV, gastric perforation, malignancy, immunosuppression

## Abstract

Herpes simplex virus (HSV) pseudotumor is a rare presentation of HSV and has not been previously reported in the stomach. A 51-year-old man with a medical history of HIV presented with new-onset dysphagia. Endoscopy revealed an HSV-positive mass at the gastroesophageal junction. After antiviral treatment, the patient returned with a 100-pound unintentional weight loss. Computed tomography showed an infiltrative mass with enlarged lymph nodes. The mass had progressed despite HSV treatment, and a repeat set of biopsies were negative for HSV with cells concerning for B-cell lymphoma. The patient was taken to the operating room for a full-thickness biopsy because of increasing concern for malignancy. The procedure was complicated by gastric perforation, leading to a total gastrectomy. Final pathology demonstrated an HSV-positive pseudotumor, negative for malignancy. It is important to diagnose gastric masses, especially in HIV-positive patients at high risk of infection and malignancy. However, immunocompromised patients with an HSV-positive mass should be treated for HSV pseudotumor with a longer than standard duration of antiviral therapy.

## INTRODUCTION

The patient is a 51-year-old man with a medical history of HIV; viral load undetectable, with CD4 count 749, on elvitegravir/cobicistat/emtricitabine/tenofovir alafenamide and darunavir; scrotal and genital herpes simplex virus (HSV); human papilloma virus lesions status post excision; and diabetes mellitus. He presented with a long-standing history of reflux treated with proton pump inhibitors and several months of dysphagia to solids and liquids. An esophagogastroduodenoscopy revealed an esophageal mass encroaching on the gastroesophageal junction circumferentially, extending from 37 to 42 cm from the incisors. Endoscopic ultrasound showed a mass in the middle and lower third of the esophagus and 3 malignant-appearing lymph nodes in the gastrohepatic ligament. The pathology showed eosinophilic esophagitis with multiple fragments of inflamed granulation tissue and fibrinopurulent exudate consistent with ulcers and rare herpes viral inclusions consistent with a herpes infection in the gastric ulcerated mass biopsy. No carcinoma or intestinal metaplasia was detected. Immunohistochemical (IHC) stains for human herpesvirus 8 and cytomegalovirus were negative, thus ruling out Kaposi sarcoma. IHC stains for HSV I/II were positive. The patient was prescribed valacyclovir 1,000 mg every 12 hours for 10 days and was treated with budesonide and proton pump inhibitors.

On repeat esophagogastroduodenoscopy performed after antiviral treatment was complete, a large, malignant-appearing mass was seen at the gastric cardia. Mass biopsies were negative for eosinophilia and negative for malignancy. The patient had an abdominal-pelvic computed tomography (CT) scan, which showed an infiltrating mass that was noted to involve the full distal esophagus, into the gastric cardia and proximal stomach with fundal sparing, seemingly much more involved than in previous endoscopies. In addition, there was an enlarged 14 mm right periesophageal lymph node. The patient was treated with bowel rest and antibiotics. He underwent peripheral flow cytometry, which was not diagnostic for a hematolymphoid malignancy.

**Figure 1.**
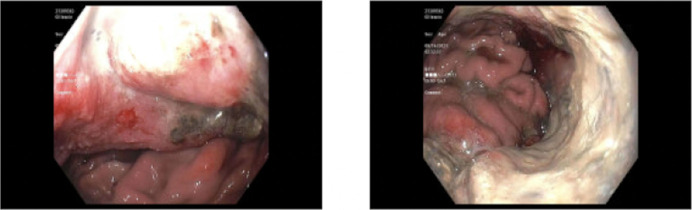
Esophagogastroduodenoscopy showing stenosis approximately 40 cm from the incisors. A large, fungating, infiltrative, ulcerated, noncircumferential mass seen in the gastric fundus and in the gastric body.

**Figure 2.**
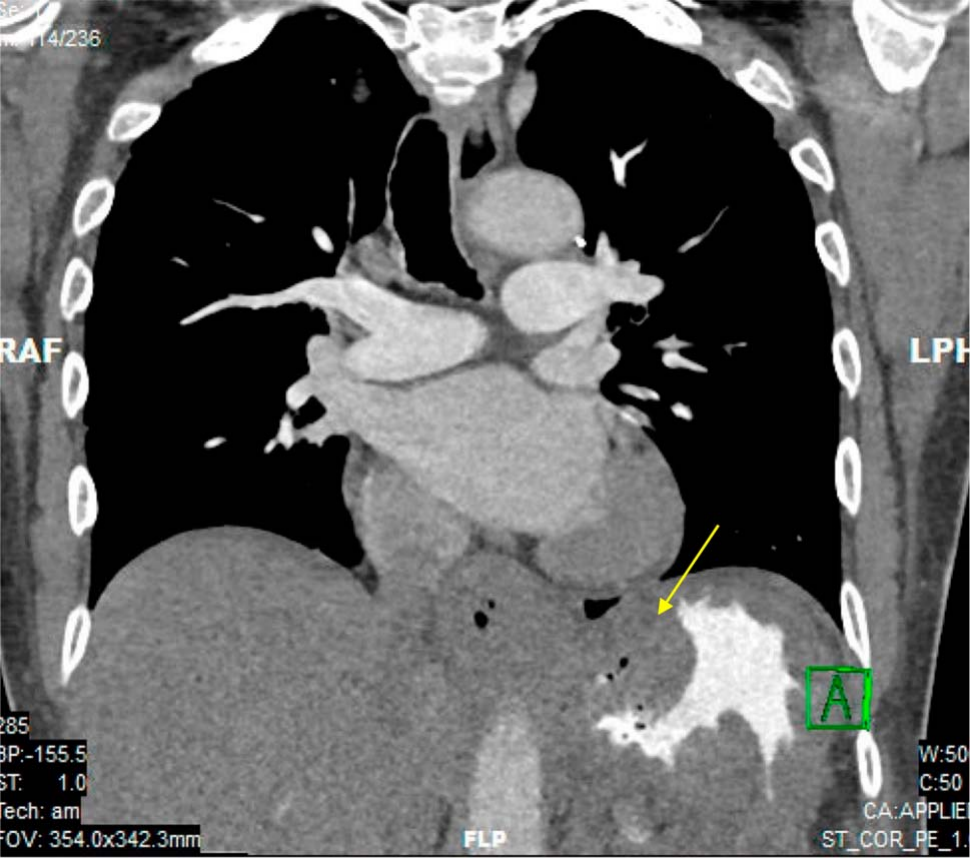
The computed tomography scan shows a thickened stomach with an infiltrating mass. This mass involves the distal esophagus, gastric cardia, and proximal stomach. The area of interest is highlighted by a yellow arrow.

After 9 months, the patient presented again because of a 100 lb unintentional weight loss. Repeat endoscopy showed a large fungating infiltrative and ulcerated noncircumferential mass in the gastric fundus and body (Figure 1). Notably, cytology from the biopsy showed an atypical Epstein-Barr virus-positive lymphoid infiltrate, suspicious for B-cell lymphoma, and IHC stains for HSV and cytomegalovirus were negative. The proliferation rate on the Ki-67 stain was 40%–50% in some areas of the sample, indicating rapid cell proliferation. Repeat abdominal-pelvic CT showed a grossly unchanged 14 mm right periesophageal lymph node and a newly enlarged gastrohepatic ligament lymph node, concerning for regional nodal metastatic disease (Figure 2). Because the patient had progression of the mass despite HSV treatment and cells concerning for lymphoma on biopsy without evidence of residual HSV on IHC staining, there was concern that biopsies performed endoscopically did not yield enough tissue for diagnosis because of the friability of the tumor. A joint decision from all contributing medical and surgical teams was made to perform a full-thickness biopsy for a definitive diagnosis to guide treatment. The biopsy was performed, and the patient initially recovered. On postoperative day 6, the patient experienced bleeding from the mass. This bleeding was initially managed with endoscopy and coiling by interventional radiology. Unfortunately, the interventions led to ischemia and gastric perforation, and the patient was taken to the operating room and underwent gastrectomy and placement of a jejunostomy tube.

**Figure 3.**
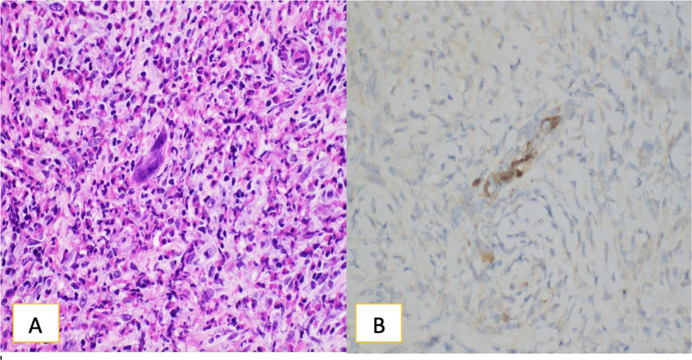
(A) H&E stain at 400× showing the distal esophageal and upper gastric mass showing extensive mixed inflammatory infiltrate rich in eosinophils and plasma cells and scattered viral inclusions with multinucleation, molding, and margination, consistent with herpes viral inclusions. (B) Immunohistochemical stain for HSV I/II highlighting the viral inclusions (brown staining). HSV, herpes simplex virus.

Pathology of the stomach showed dense fibrotic connective tissue with extensive transmural chronic mixed inflammatory infiltrate, composed predominantly of eosinophils, plasma cells, and histiocytes, with scattered lymphocytes that had rare herpes viral inclusions confirmed by immunohistochemical stains for HSV I/II and polymerase chain reaction testing (Figure 3). Immunohistochemical stains for kappa and lambda light chains showed no definite evidence of light chain restriction, decreasing the likelihood of a plasma cell disorder. The patient was treated for HSV with a 21-day course of acyclovir (5 mg/kg IV every 8 hours). The patient has since recovered from surgery, is gaining weight, and is tolerating a regular per os (PO) diet.

## DISCUSSION

HSV is a rare cause of gastric masses with few documented cases.^1–3^ HSV infection of the esophagus and stomach usually presents in immunocompromised patients, especially in those who are HIV-positive.^1–3^ This HSV infection can present with odynophagia or dysphagia, and a few reports describe an association with eosinophilic esophagitis, all of which were observed in this patient.^2,4,5^ HSV lesions usually affect the squamous mucosa of the distal esophagus and present as a vesicle, ulcer, or plaque. However, HSV pseudotumors mimic other malignancies, which is of great concern in HIV-positive or other immunocompromised populations with a significantly higher risk of lymphoma than the general population.^6^

A few case reports show the presentation of HSV with an extensive inflammatory response and mass-like lesion. Two of these cases include patients with HSV in the hypopharynx and in the distal colon and rectum.^3,7–10^ In one case report, a patient with a medical history of HIV presented with an ulcerative mass on the hypopharynx.^8^ Initially, the mass was believed to be malignant, and the patient underwent multiple biopsies and surgeries over 19 months. This treatment did not lead to the resolution of the mass. Biopsy and cytopathology showed HSV, which was subsequently resolved with valacyclovir 1,000 mg every 12 hours for 4 weeks. Repeat visualization showed a decrease in the size of the mass and increased visualization and patency of the esophagus. Another case report by Bai et al illustrated a patient who was HIV-positive and presented with a perforated and necrotic rectal mass with enlarged mesorectal lymph nodes. The patient was experiencing obstructive symptoms and required diversion with a sigmoid colostomy. A biopsy showed positive HSV1 and HSV2 IHC stains, and the patient was treated with oral valacyclovir 1,000 mg every 12 hours for 3 weeks and rectal symptoms resolved after the treatment.^7^ These cases have many similarities with our patient's presentation and may indicate the need for longer, suppressive antiviral treatment.

Owing to the rarity of HSV pseudotumors, treatment guidelines are extrapolated from the HSV esophagitis literature detailed in this discussion. A review of 4 case reports of patients with herpes simplex esophagitis reported that therapy with acyclovir may attenuate infection and hasten symptom resolution, although HSV esophagitis is often self-limiting in an immunocompetent patient.^11^ Although there are no clear guidelines on the treatment of HSV esophagitis and HSV pseudotumor, published case reports and UpToDate have recommended treatment of HSV esophagitis for 7–10 days in immunocompetent patients and 14–21 days in immunocompromised patients.^2,7,8,12,13^ The treatment of HSV esophagitis in an immunocompetent patient is acyclovir (200 mg PO 5 times a day or 400 mg PO 3 times a day for 7–10 days).^2^ The treatment of HSV esophagitis in an immunocompromised patient includes acyclovir 400 mg PO 5 times a day for 2–3 weeks.^2,14^ This extended dose duration for immunocompromised patients is because of HSV’s resistance to acyclovir.^4,15^

Our patient's lesion did not present as a typical HSV lesion, and instead, it presented as an inflammatory pseudotumor with atypical Epstein-Barr virus-positive lymphoid infiltrate, which raised suspicion for B-cell lymphoma. This suspicion was increased when the mass was larger on repeat evaluation after antiviral treatment and biopsies were negative for HSV. This suspicion was also elevated because of the patient's HIV status and regional lymphadenopathy seen on a CT scan. Some case reports mentioned that the HSV pseudotumor presented with regional lymphadenopathy.^7,8^ The presence or absence of this finding may be important to characterize because regional lymphadenopathy may support HSV pseudotumor on the differential instead of what medical professionals would usually assume is regional nodal metastatic disease.

Based on the patient's presentation and our course of treatment, we believe that it is important to adequately treat patients for HSV, especially in immunocompromised patients or patients with a history of HIV. Initially, we treated the patient with a 10-day course of valacyclovir because of an undetectable HIV viral load and normal CD4 count, leading us to believe that he was immunocompetent. However, the clinical course was not adequate for our patient despite negative IHC results. On review of the case and the persistence of HSV after treatment, we believe a longer clinical course may have helped the patient symptomatically and possibly have avoided surgery and prolonged symptoms. Because there are no definitive treatment guidelines, it is important to add to the limited existing literature on HSV pseudotumor treatment and the potential benefit of an extended course of treatment.

Through our case, we aim to highlight that HSV pseudotumor should be on the differential diagnosis when evaluating masses with regional lymphadenopathy in immunocompromised patients. HSV pseudotumors are uncommon and may present as mass-like lesions, which are concerning for malignancy. This is especially concerning in patients who are immunocompromised, such as our patient with HIV, when missing an infection or malignancy can have serious consequences. Furthermore, we wish to add to the literature on treating HSV pseudotumor by highlighting the need for an extended antiviral course in patients who may be immunocompromised.

## DISCLOSURES

Author contributions: All authors were involved in substantially contributing to the conception and design of the work. S. Kripalani, J. Williams, and P. Bansal drafted the manuscript, and all authors partook in editing and proofreading the manuscript. U. Joneja helped with providing the team with pathology slides. P. Bansal provided additional infectious disease perspective on the topic. All authors agreed on the final approval of the version to be submitted. F. Spitz is the article guarantor.

Financial disclosure: None to report.

Previous presentation: This case report was initially presented at Camden Scholars Forum; Presented April 2022; Camden, NJ (Virtual Poster Presentation).

Informed consent was obtained for this case report.
